# Supplementary material for: Characteristics of a transgender and gender-diverse patient population in Utah: Use of electronic health records to advance clinical and health equity research
Source: PLoS One. 2024 May 7;19(5):e0302895. doi: 10.1371/journal.pone.0302895 (PMC11075904; doi:10.1371/journal.pone.0302895)
Supplement: S1 Table — (DOCX) [file pone.0302895.s001.docx]

Supplemental Table 1: List of estrogen- and testosterone- based medications included

| Estrogen-Based | Testosterone-Based |
| --- | --- |
| \| CLIMARA 0.05 MG/24HR TD PTWK \| \| --- \| \| CLIMARA 0.1 MG/24HR TD PTWK \| \| DELESTROGEN 10 MG/ML IM OIL \| \| DELESTROGEN 20 MG/ML IM OIL \| \| DELESTROGEN IM \| \| DEPO-ESTRADIOL 5 MG/ML IM OIL \| \| DEPO-ESTRADIOL IM \| \| DOTTI 0.025 MG/24HR TD PTTW \| \| DOTTI 0.1 MG/24HR TD PTTW \| \| EC-RX ESTRADIOL 0.4 % TD CREA \| \| ENJUVIA 1.25 MG PO TABS \| \| ESTERIFIED ESTROGENS 1.25 MG PO TABS \| \| ESTERIFIED ESTROGENS PO \| \| ESTRACE 1 MG OR TABS \| \| ESTRACE 1 MG PO TABS \| \| ESTRACE 2 MG OR TABS \| \| ESTRACE 2 MG PO TABS \| \| ESTRADIOL 0.025 MG/24HR TD PTTW \| \| ESTRADIOL 0.025 MG/24HR TD PTWK \| \| ESTRADIOL 0.0375 MG/24HR TD PTTW \| \| ESTRADIOL 0.0375 MG/24HR TD PTWK \| \| ESTRADIOL 0.05 MG/24HR TD PTTW \| \| ESTRADIOL 0.05 MG/24HR TD PTWK \| \| ESTRADIOL 0.06 MG/24HR TD PTWK \| \| ESTRADIOL 0.075 MG/24HR TD PTTW \| \| ESTRADIOL 0.075 MG/24HR TD PTWK \| \| ESTRADIOL 0.1 MG/24HR TD PTTW \| \| ESTRADIOL 0.1 MG/24HR TD PTWK \| \| ESTRADIOL 0.25 MG/0.25GM TD GEL \| \| ESTRADIOL 0.5 MG PO HALF TAB \| \| ESTRADIOL 0.5 MG PO TABLET \| \| ESTRADIOL 0.5 MG PO TABS \| \| ESTRADIOL 0.5 MG/0.5GM TD GEL \| \| ESTRADIOL 1 MG OR TABS \| \| ESTRADIOL 1 MG PO TABLET \| \| ESTRADIOL 1 MG PO TABS \| \| ESTRADIOL 1 MG SL TABLET (TRANSGENDER CARE ONLY) \| \| ESTRADIOL 1 MG/GM TD GEL \| \| ESTRADIOL 1.5 MG PO TABS \| \| ESTRADIOL 10 MG IL PLLT \| \| ESTRADIOL 2 MG OR TABS \| \| ESTRADIOL 2 MG PO TABLET \| \| ESTRADIOL 2 MG PO TABS \| \| ESTRADIOL 2 MG SL TABLET (TRANSGENDER CARE ONLY) \| \| ESTRADIOL 20 MG IL PLLT \| \| ESTRADIOL 6 MG IL PLLT \| \| ESTRADIOL ACETATE PO \| \| ESTRADIOL CYPIONATE 5 MG/ML IM OIL \| \| ESTRADIOL CYPIONATE IM \| \| ESTRADIOL IL \| \| ESTRADIOL PO \| \| ESTRADIOL VALERATE 10 MG/ML IM OIL \| \| ESTRADIOL VALERATE 20 MG/ML IM OIL \| \| ESTRADIOL VALERATE 40 MG/ML IM OIL \| \| ESTRADIOL VALERATE IM \| \| ESTROGENS CONJ SYNTHETIC A 1.25 MG PO TABS \| \| ESTROGENS CONJ SYNTHETIC A PO \| \| ESTROGENS CONJ SYNTHETIC B 0.45 MG PO TABS \| \| ESTROGENS CONJ SYNTHETIC B 0.625 MG OR TABS \| \| ESTROGENS CONJ SYNTHETIC B 1.25 MG PO TABS \| \| ESTROGENS CONJ SYNTHETIC B PO \| \| ESTROGENS CONJUGATED 0.3 MG PO TABLET \| \| ESTROGENS CONJUGATED 0.3 MG PO TABS \| \| ESTROGENS CONJUGATED 0.625 MG PO TABLET \| \| ESTROGENS CONJUGATED 0.625 MG PO TABS \| \| ESTROGENS CONJUGATED 0.9 MG PO TABLET \| \| ESTROGENS CONJUGATED 0.9 MG PO TABS \| \| ESTROGENS CONJUGATED 1.25 MG PO TABLET \| \| ESTROGENS CONJUGATED 1.25 MG PO TABS \| \| ESTROGENS CONJUGATED 25 MG IJ SOLR \| \| ESTROGENS CONJUGATED IJ \| \| ESTROGENS CONJUGATED PO \| \| LYLLANA 0.1 MG/24HR TD PTTW \| \| MINIVELLE TD \| \| PREMARIN 0.625 MG OR TABS \| \| PREMARIN 0.625 MG PO TABLET \| \| PREMARIN 1.25 MG OR TABS \| \| PREMARIN 1.25 MG PO TABLET \| \| PREMARIN 1.25 MG PO TABS \| \| PREMARIN 2.5 MG OR TABS \| \| PREMARIN PO \| \| VIVELLE-DOT TD \| | \| C TESTOSTERONE 20 % CREAM 200 MG/ML \| \| --- \| \| DEPO-TESTOSTERONE 100 MG/ML IM SOLN \| \| DEPO-TESTOSTERONE 200 MG/ML IM OIL \| \| DEPO-TESTOSTERONE 200 MG/ML IM SOLN \| \| DEPO-TESTOSTERONE IM \| \| FIRST-TESTOSTERONE MC TD \| \| FIRST-TESTOSTERONE TD \| \| TESTOSTERONE 1 MG/0.02 ML - COMPOUNDED CREAM \| \| TESTOSTERONE 1 MG/0.2 ML - COMPOUNDED CREAM \| \| TESTOSTERONE 1.25 GM/ACT (1%) TD GEL \| \| TESTOSTERONE 1.62 % TD GEL \| \| TESTOSTERONE 1.62 % TD GEL \| \| TESTOSTERONE 10 MG/ACT (2%) TD GEL \| \| TESTOSTERONE 100 MG IL PLLT \| \| TESTOSTERONE 12.5 MG IL PLLT \| \| TESTOSTERONE 12.5 MG/ACT (1%) TD GEL \| \| TESTOSTERONE 2 MG/24HR TD PT24 \| \| TESTOSTERONE 20.25 MG/1.25GM (1.62%) TD GEL \| \| TESTOSTERONE 20.25 MG/ACT (1.62%) TD GEL \| \| TESTOSTERONE 200 MG IL PLLT \| \| TESTOSTERONE 25 MG IL PLLT \| \| TESTOSTERONE 25 MG/2.5GM (1%) TD GEL \| \| TESTOSTERONE 25 MG/2.5GM TD GEL \| \| TESTOSTERONE 30 MG BU MISC \| \| TESTOSTERONE 30 MG/ACT TD SOLN \| \| TESTOSTERONE 4 MG/24HR TD PT24 \| \| TESTOSTERONE 40.5 MG/2.5GM (1.62%) TD GEL \| \| TESTOSTERONE 5 MG/24HR TD PT24 \| \| TESTOSTERONE 50 MG/5GM (1%) TD GEL \| \| TESTOSTERONE 50 MG/5GM TD GEL \| \| TESTOSTERONE 75 MG IL PLLT \| \| TESTOSTERONE 87.5 MG IL PLLT \| \| TESTOSTERONE CYPIONATE & PROP IJ \| \| TESTOSTERONE CYPIONATE & PROP IM \| \| TESTOSTERONE CYPIONATE 100 MG/ML IJ SOLN \| \| TESTOSTERONE CYPIONATE 100 MG/ML IM OIL \| \| TESTOSTERONE CYPIONATE 100 MG/ML IM SOLN \| \| TESTOSTERONE CYPIONATE 100 MG/ML SC SOLN \| \| TESTOSTERONE CYPIONATE 150 MG/ML IJ SOLN \| \| TESTOSTERONE CYPIONATE 200 MG/ML IJ SOLN \| \| TESTOSTERONE CYPIONATE 200 MG/ML IM KIT \| \| TESTOSTERONE CYPIONATE 200 MG/ML IM OIL \| \| TESTOSTERONE CYPIONATE 200 MG/ML IM SOLN \| \| TESTOSTERONE CYPIONATE 200 MG/ML SC SOLN \| \| TESTOSTERONE CYPIONATE 250 MG/ML IM SOLN \| \| TESTOSTERONE CYPIONATE 50 MG/ML IJ SOLN \| \| TESTOSTERONE CYPIONATE IJ \| \| TESTOSTERONE CYPIONATE IM \| \| TESTOSTERONE ENANTHATE 100 MG/0.5ML SC SOAJ \| \| TESTOSTERONE ENANTHATE 200 MG/ML IJ SOLN \| \| TESTOSTERONE ENANTHATE 200 MG/ML IM OIL \| \| TESTOSTERONE ENANTHATE 200 MG/ML IM SOLN \| \| TESTOSTERONE ENANTHATE 50 MG/0.5ML SC SOAJ \| \| TESTOSTERONE ENANTHATE 75 MG/0.5ML SC SOAJ \| \| TESTOSTERONE ENANTHATE IJ \| \| TESTOSTERONE ENANTHATE IM \| \| TESTOSTERONE ENANTHATE SC \| \| TESTOSTERONE GEL 100 MG/GM (COMPOUND) \| \| TESTOSTERONE GEL 50 MG/GM (COMPOUND) \| \| TESTOSTERONE IL \| \| TESTOSTERONE OINTMENT 5% \| \| TESTOSTERONE PROPIONATE IJ \| \| TESTOSTERONE TD \| \| TESTOSTERONE TOPICAL CREAM ERX CMPD \| \| TESTOSTERONE UNDECANOATE 750 MG/3ML IM SOLN \| \| TESTOSTERONE UNDECANOATE IM \| |
