# Supplementary material for: Characteristics of a transgender and gender-diverse patient population in Utah: Use of electronic health records to advance clinical and health equity research
Source: PLoS One. 2024 May 7;19(5):e0302895. doi: 10.1371/journal.pone.0302895 (PMC11075904; doi:10.1371/journal.pone.0302895)
Supplement: Attachment — Submitted filename: PONE-D-23-40640_reviewer_comments.pdf [file pone.0302895.s002.pdf]

PLOS ONE

Characteristics of a transgender and gender-diverse patient population in Utah: Use of electronic health records to advance clinical and health equity research

--Manuscript Draft--

Manuscript Number:	PONE-D-23-40640
Article Type:	Research Article
Full Title:	Characteristics of a transgender and gender-diverse patient population in Utah: Use of electronic health records to advance clinical and health equity research
Short Title:	Using electronic health records to identify transgender and gender diverse adults
Corresponding Author:	TIFFANY HO, MD, MPH<br>The University of Utah School of Medicine<br>Salt Lake City, UT UNITED STATES
Keywords:	Transgender Care; Sexual and Gender Minority; gender identity; Gender-Affirming Care; Gender-Affirming Hormone Therapy; Gender Affirming Surgery; Electronic Health Record; administrative data; Retrospective Cohort Analysis; Health Equity
Abstract:	Transgender and gender-diverse (TGD) people, individuals whose gender identity differs from their sex assigned at birth, face unique challenges in accessing gender-affirming care and often experience disparities in a variety of health outcomes. Clinical research on TGD health is limited by a lack of standardization on how to best identify these individuals. The objective of this retrospective cohort analysis was to accurately identify and describe TGD adults and their use of gender-affirming care from 2003 – 2023 in a healthcare system in Utah, United States. International Classification of Disease (ICD)-9 and 10 codes and surgical procedure codes, along with sexual orientation and gender identity data were used to develop a dataset of 4,764 TGD adults. During this time frame, 2,995 adults have received gender-affirming hormone therapy (GAHT) and/or gender-affirming surgery (GAS) within one healthcare system. There was no significant difference in race or ethnicity between TGD adults who received GAHT and/or GAS compared to TGD adults who did not receive such care. TGD adults who received GAHT and/or GAS were more likely to have commercial insurance coverage, and adults from rural communities were underrepresented. Patients seeking estradiol-based GAHT tended to be older than those seeking testosterone-based GAHT. The first GAS occurred in 2013, and uptake of GAS have doubled since 2018. This study provides a methodology to identify and examine TGD patients in other health systems and offers insights into emerging trends and access to gender-affirming care.
Order of Authors:	TIFFANY HO, MD, MPH
	Brian Zenger, MD, PhD
	Bayarmaa Mark, PhD
	Laurel Hiatt
	Erika Sullivan, MD, MS, MS
	Benjamin A Steinberg, MD, MHS, FACP, FACC, FHRS
	Ann Lyons
	Adam M Spivak
	Cori Agarwal
	Marisa Adelman
	James Hotaling
	Bernadette Kiraly
	Sharon Talboys
Additional Information:	

Powered by Editorial Manager® and ProduXion Manager® from Aries Systems Corporation

Question	Response
**Financial Disclosure**<br><br>Enter a financial disclosure statement that describes the sources of funding for the work included in this submission. Review the submission guidelines for detailed requirements. View published research articles from *PLOS ONE* for specific examples.<br><br>This statement is required for submission and **will appear in the published article** if the submission is accepted. Please make sure it is accurate.<br><br>**Funded studies**<br>Enter a statement with the following details:<br>• Initials of the authors who received each award<br>• Grant numbers awarded to each author<br>• The full name of each funder<br>• URL of each funder website<br>• Did the sponsors or funders play any role in the study design, data collection and analysis, decision to publish, or preparation of the manuscript?<br><br>Did you receive funding for this work?	Yes
Please add funding details.<br>as follow-up to "**Financial Disclosure**<br><br>Enter a financial disclosure statement that describes the sources of funding for the work included in this submission. Review the submission guidelines for detailed requirements. View published research articles from *PLOS ONE* for specific examples.<br><br>This statement is required for submission and **will appear in the published article** if the submission is accepted. Please make sure it is accurate.	(ES and TH) This study was supported by the University of Utah Department of Family and Preventive Medicine, Health Studies Fund, Research Pilot Award. The funders had no role in study design, data collection and analysis, decision to publish, or preparation of the manuscript.<br><br>(BZ) This study was partially supported by the National Institute of Health (NIH) National Heart, Lung, and Blood Institute (NHLBI) Grant no. 1F30HL149327 https://www.nhlbi.nih.gov/ The funders had no role in study design, data collection and analysis, decision to publish, or preparation of the manuscript.

**Funded studies**<br>Enter a statement with the following details:<br>• Initials of the authors who received each award<br>• Grant numbers awarded to each author<br>• The full name of each funder<br>• URL of each funder website<br>• Did the sponsors or funders play any role in the study design, data collection and analysis, decision to publish, or preparation of the manuscript?<br><br>Did you receive funding for this work?"	
Please select the country of your main research funder (please select carefully as in some cases this is used in fee calculation).<br>as follow-up to "**Financial Disclosure**<br><br>Enter a financial disclosure statement that describes the sources of funding for the work included in this submission. Review the submission guidelines for detailed requirements. View published research articles from *PLOS ONE* for specific examples.<br><br>This statement is required for submission and **will appear in the published article** if the submission is accepted. Please make sure it is accurate.<br><br>**Funded studies**<br>Enter a statement with the following details:<br>• Initials of the authors who received each award<br>• Grant numbers awarded to each author<br>• The full name of each funder<br>• URL of each funder website<br>• Did the sponsors or funders play any role in the study design, data collection and analysis, decision to publish, or preparation of the manuscript?<br><br>Did you receive funding for this work?"	UNITED STATES - US
**Competing Interests**	The authors have declared that no competing interests exist.

Use the instructions below to enter a competing interest statement for this submission. On behalf of all authors, disclose any competing interests that could be perceived to bias this work—acknowledging all financial support and any other relevant financial or non-financial competing interests.<br><br>This statement is required for submission and **will appear in the published article** if the submission is accepted. Please make sure it is accurate and that any funding sources listed in your Funding Information later in the submission form are also declared in your Financial Disclosure statement.<br><br>View published research articles from *PLOS ONE* for specific examples.<br><br>**NO authors have competing interests**<br><br>Enter: *The authors have declared that no competing interests exist.*<br><br>**Authors with competing interests**<br><br>Enter competing interest details beginning with this statement:<br><br>*I have read the journal's policy and the authors of this manuscript have the following competing interests: [insert competing interests here]*<br><br>* typeset	
**Ethics Statement**<br><br>Enter an ethics statement for this submission. This statement is required if the study involved:<br><br>• Human participants<br>• Human specimens or tissue<br>• Vertebrate animals or cephalopods<br>• Vertebrate embryos or tissues	Approved by the University of Utah Institutional Review Board (Umbrella IRB_00161271)<br>Consent was not obtained as this was a retrospective chart review, participants were not contacted for this study and data was analyzed anonymously

• Field research<br><br>Write "N/A" if the submission does not require an ethics statement.<br><br>General guidance is provided below. Consult the submission guidelines for detailed instructions. **Make sure that all information entered here is included in the Methods section of the manuscript.**	

**Format for specific study types**<br><br>**Human Subject Research (involving human participants and/or tissue)**<br>• Give the name of the institutional review board or ethics committee that approved the study<br>• Include the approval number and/or a statement indicating approval of this research<br>• Indicate the form of consent obtained (written/oral) or the reason that consent was not obtained (e.g. the data were analyzed anonymously)<br><br>**Animal Research (involving vertebrate animals, embryos or tissues)**<br>• Provide the name of the Institutional Animal Care and Use Committee (IACUC) or other relevant ethics board that reviewed the study protocol, and indicate whether they approved this research or granted a formal waiver of ethical approval<br>• Include an approval number if one was obtained<br>• If the study involved *non-human primates*, add *additional details* about animal welfare and steps taken to ameliorate suffering<br>• If anesthesia, euthanasia, or any kind of animal sacrifice is part of the study, include briefly which substances and/or methods were applied<br><br>**Field Research**<br><br>Include the following details if this study involves the collection of plant, animal, or other materials from a natural setting:<br>• Field permit number<br>• Name of the institution or relevant body that granted permission	
**Data Availability**<br><br>Authors are required to make all data underlying the findings described fully available, without restriction, and from the time of publication. PLOS allows rare exceptions to address legal and ethical concerns. See the PLOS Data Policy and FAQ for detailed information.	No - some restrictions will apply

Powered by Editorial Manager® and ProduXion Manager® from Aries Systems Corporation

A Data Availability Statement describing where the data can be found is required at submission. Your answers to this question constitute the Data Availability Statement and **will be published in the article**, if accepted.<br><br>**Important:** Stating 'data available on request from the author' is not sufficient. If your data are only available upon request, select 'No' for the first question and explain your exceptional situation in the text box.<br><br>Do the authors confirm that all data underlying the findings described in their manuscript are fully available without restriction?	
**Describe where the data may be found in full sentences. If you are copying our sample text, replace any instances of XXX with the appropriate details.**<br><br>• If the data are **held or will be held in a public repository**, include URLs, accession numbers or DOIs. If this information will only be available after acceptance, indicate this by ticking the box below. For example: *All XXX files are available from the XXX database (accession number(s) XXX, XXX.*).<br>• If the data are all contained **within the manuscript and/or Supporting Information files**, enter the following: *All relevant data are within the manuscript and its Supporting Information files.*<br>• If neither of these applies but you are able to provide **details of access elsewhere**, with or without limitations, please do so. For example:<br><br>*Data cannot be shared publicly because of [XXX]. Data are available from the XXX Institutional Data Access / Ethics Committee (contact via XXX) for researchers who meet the criteria for access to confidential data.*<br><br>*The data underlying the results presented in the study are available from (include the name of the third party*	The information will only be available after acceptance.<br><br>Data cannot be shared publicly because of concern for current legislation in the state of Utah that may ban gender-affirming care and they may utilize this information to target specific individuals (the dataset uses zip code which can be identifiable). Data are available from the University of Utah Institutional Data Access / Ethics Committee (contact via corresponding author Tiffany Ho) for researchers who meet the criteria for access to confidential data.

and contact information or URL).<br>• This text is appropriate if the data are owned by a third party and authors do not have permission to share the data.<br><br>* typeset	
Additional data availability information:	Tick here if the URLs/accession numbers/DOIs will be available only after acceptance of the manuscript for publication so that we can ensure their inclusion before publication.

Powered by Editorial Manager® and ProduXion Manager® from Aries Systems Corporation

Manuscript - Updated IRB
Click here to access/download;Manuscript;Transgender Cohort Paper 12.19.23 updated IRB.docx

Characteristics of a transgender and gender-diverse patient population in Utah: Use of electronic health records to advance clinical and health equity research

Tiffany Ho[1*], Brian Zenger[2], Bayarmaa Mark[1], Laurel Hiatt[3], Erika Sullivan[1], Benjamin A. Steinberg[4], Ann Lyons[5], Adam M. Spivak[6], Cori Agarwal[7], Marisa Adelman[8], James Hotaling[9], Bernadette Kiraly[1], Sharon Talboys[1]

[1] Department of Family and Preventive Medicine, University of Utah School of Medicine, Salt Lake City, Utah, United States of America

[2] Department of Internal Medicine, Washington University in St. Louis, St. Louis, Missouri, United States of America

[3] Department of Human Genetics, University of Utah, Salt Lake City, Utah, United States of America

[4] Division of Cardiology, University of Utah School of Medicine, Salt Lake City, Utah, United States of America

[5] Data Science Services, University of Utah, Salt Lake City, Utah, United States of America

[6] Division of Infectious Diseases, University of Utah School of Medicine, Salt Lake City, Utah, United States of America

[7] Division of Plastic Surgery, Department of Surgery, University of Utah, Salt Lake City, Utah, United States of America

[8] Department of Obstetrics & Gynecology, University of Utah, Salt Lake City, Utah, United States of America

[9] Division of Urology, Department of Surgery, University of Utah, Salt Lake City, Utah, United States of America

* Corresponding author

Email: tiffany.ho@hsc.utah.edu (TH)

Abstract

Transgender and gender-diverse (TGD) people, individuals whose gender identity differs from their sex assigned at birth, face unique challenges in accessing gender-affirming care and often experience disparities in a variety of health outcomes. Clinical research on TGD health is limited by a lack of standardization on how to best identify these individuals. The objective of this retrospective cohort analysis was to accurately identify and describe TGD adults and their use of gender-affirming care from 2003 – 2023 in a healthcare system in Utah, United States. International Classification of Disease (ICD)-9 and 10 codes and surgical procedure codes, along with sexual orientation and gender identity data were used to develop a dataset of 4,764 TGD adults. During this time frame, 2,995 adults have received gender-affirming hormone therapy (GAHT) and/or gender-affirming surgery (GAS) within one healthcare system. There was no significant difference in race or ethnicity between TGD adults who received GAHT and/or GAS compared to TGD adults who did not receive such care. TGD adults who received GAHT and/or GAS were more likely to have commercial insurance coverage, and adults from rural communities were underrepresented. Patients seeking estradiol-based GAHT tended to be older than those seeking testosterone-based GAHT. The first GAS occurred in 2013, and uptake of GAS have doubled since 2018. This study provides a methodology to identify and examine TGD patients in other health systems and offers insights into emerging trends and access to gender-affirming care.

Introduction

In recent years, there is increasing recognition of the unique healthcare needs of transgender and gender-diverse (TGD) individuals. In the World Professional Association of Transgender Health (WPATH) Standards of Care for Transgender and Gender Diverse People, Version 8 [1] offers a broad and comprehensive description of people with gender identities or expressions that differ from the gender socially attributed to their sex assigned to them at birth. Estimates of the size of this population vary, but recent studies suggest that approximately 0.6-1% of the general population identifies as TGD [2–4]. As this population seeks healthcare services, it is imperative that research focuses on addressing their specific needs to provide effective, evidence-based care.

Based on small studies with limited datasets, the TGD community experiences more discrimination in the healthcare system than the general population [5,6], and this stigma drives health disparities [5,7]. Transgender and gender-diverse people have higher rates of mental health conditions (anxiety, depression, suicidality, post-traumatic stress disorder) [8–10], substance use disorder [11,12], infectious diseases including HIV/AIDS [13–15] among many other health concerns. These health disparities arise from a complex interplay of factors, including minority stress, stigma, discrimination, barriers to accessing care, and a lack of provider knowledge and cultural competency [7,16–18]. Many studies on this population rely on small sample sizes [19–21], convenience sampling [2,22–24], or large administrative databases with limited clinical detail [4,25,26] resulting in limited generalizability of findings. Moreover, most of the TGD health research to date has been qualitative or cross-sectional in nature, lacking longitudinal data that could provide insights into the long-term health outcomes and care trajectories of TGD adults.

Given the increased visibility of TGD experience in the past ten years, it is of critical importance to identify the health needs of TGD individuals particularly in the lens of medical management. The objectives of this study were to characterize the community of TGD adults who received clinical care at a large healthcare system in Utah and to explore the rates of TGD adults who receive gender-affirming hormone-therapy (GAHT) and/or gender-affirming surgeries (GAS). The goal of this manuscript is to share the methodology of creating this cohort so others can apply and improve upon this method to advance understanding of important clinical and health equity questions in the field of transgender health.

Methods

This retrospective cohort study examined clinical encounter data from a Utah-based healthcare system for individuals over the age of 18 years old seeking gender-affirming care. Clinical and administrative billing diagnosis encounters between 2003 –April 2023 were used to determine the base cohort. Inpatient, outpatient, and procedural visits, as well as medication orders and laboratory results relevant to gender-affirming care were also retrieved. The data was derived from the health system's enterprise data warehouse upon approval by the University of Utah's Institutional Review Board (IRB_00159449).

The diagnostic International Classification of Disease (ICD) version 9 and 10 codes specific for "gender dysphoria," and TGD adults were selected based on methodology described in earlier studies [27–30]. Adults aged 18 years and older were included in the dataset if they had at least one clinical encounter that billed specific codes commonly associated with TGD individuals (table 1).

Table 1: ICD-9 and ICD-10 codes used to identify potentially eligible transgender and gender-diverse adults for cohort

ICD-9 Diagnostic Code		ICD-10 Diagnostic Code	
301.50	Trans-sexualism with unspecified sexual history (aka ‘trans-sexualism not otherwise specified’)	F64.0	Transsexualism
302.51	Trans-sexualism with asexual history	F64.1	Dual role transvestism
302.52	Trans-sexualism with homosexual history	F64.2	Gender identity disorder of childhood
302.53	Trans-sexualism with heterosexual history	F64.8	Other gender identity disorders
302.6	Gender identity disorder in children	F64.9	Gender identity disorder unspecified
302.85	Gender identity disorder in adolescents or adults	Z87.890	Personal history of sex reassignment

Surgical procedures were identified based on the Current Procedural Terminology (CPT) codes associated with GAS as listed in table 2. These codes were collected from both literature review [31,32] and from the surgeons who performed gender-affirming surgeries within one Utah-based healthcare system.

Table 2: Current Procedural Terminology (CPT) codes used for gender-affirming surgeries

Gender-Affirming Surgical Procedures	Top	Bottom
Feminizing	*Chest Wall Reconstruction (breast augmentation)*<br><br>19325	*Orchiectomy* [32]<br><br>54520,54521,54522,54530,54535<br><br>*Vaginoplasty*<br><br>53420, 53430, 54125, 54520, 54690, 55970, 56800, 56805, 57291, 57292, 57335

Masculinizing	*Chest Wall Reconstruction (bilateral mastectomies)*<br><br>19300,19301,19303,19304, 19318	*Phalloplasty*<br><br>53425, 54660, 55175, 55180, 55980, 57106, 57110<br><br>*Oophorectomy*<br><br>57531, 58200, 58210, 58240, 58240, 58548, 58943, 58950, 58951, 58952, 58953, 58954, 58956, 58291, 58542, 58544, 58548, 58552, 58554, 58571, 58573, 58951, 58953, 58954, 58956, 58700<br><br>*Hysterectomy* [31]<br><br>58260, 58285, 58290, 58541, 58543, 58550, 58553, 58570, 5857, 58291, 58542, 58544, 58548, 58552, 58554, 58571, 58573, 58951, 58953, 58954, 58956, 56307, 56308, 58150, 58152, 58180, 58200, 58210

The accuracy of the TGD cohort was verified via a random sample of 50 patients selected based on ICD codes with 100% accuracy. Furthermore, a random sample of 50 known TGD adults were selected from an outpatient clinic list and 100% of the known TGD adults appeared in the ICD based selection. Due to discordance in the type of hormone therapy, gender-affirming surgery and/or gender identity, manual chart review was completed for 101 TGD adults in the GAHT group and 22 were excluded as they did not meet the criteria for TGD. Thirty-nine adults had prescriptions for both testosterone- and estrogen-based therapy. Of those, five were excluded as they did not meet the criteria for TGD (all postmenopausal cis-women on hormone replacement therapy), four adults had detransitioned back to their natal sex, and one adult had

active prescriptions for both injectable testosterone and oral estradiol. To reduce mis-categorization for gender-affirming surgeries, manual chart review was performed for 93 adults who had surgery types that were either incongruent with each other or with the category of GAHT (e.g. individual with hysterectomy who was also on estrogen due to endometrial cancer). Thirteen adults from the GAS group were excluded as they did not meet the criteria for TGD.

Metrics

Transgender and gender-diverse adults were categorized into three groups: those who underwent GAS; those actively managed on GAHT; and those who had the ICD-9 or -10 diagnosis of gender dysphoria but had not undergone active medical therapy or surgical intervention (not actively managed). The specific inclusion criteria for each group are shown in table 3. Of note, the first two groups are not exclusive of each other. Adults who had any prescription for GAHT were categorized as estrogen-based or testosterone-based hormone therapy (supplemental table 1). The type of hormone was used to define the broader categories instead of transfeminine and transmasculine to be inclusive of non-binary and gender-diverse adults. Considering that some of these individuals may be receiving active hormone therapy outside of our healthcare system, adults were not included in the actively managed group if they had less than two prescriptions at different dates confirmed within the health system. Thus, adults with two or more unique prescriptions were considered actively managed for GAHT (Figure 1).

Table 3. Inclusion Criteria for Gender Affirming Hormone Therapy (GAHT) and Gender-Affirming Surgery Groups

Study Group	Inclusion Criteria

General Testosterone-based GAHT	Adults with at least one prescription for testosterone
General Estrogen-based GAHT	Adults with at least one prescription for estrogen
Actively Managed Testosterone-based GAHT	Two or more prescriptions of testosterone, each at least one day apart
Active Managed Estrogen-based GAHT	Two or more prescriptions of estrogen, each at least one day apart
Gender-Affirming Surgery	Any gender affirming surgical interventions as defined in supplemental table 1
Not Actively Managed	Adults with the ICD-9 or ICD-10 code who have not received any medical therapy or surgical intervention. Includes adults who had less than 2 prescriptions of estrogen- or testosterone-based GAHT

The index visit for the cohort was defined as the date of the first prescription of GAHT prescribed in the health care system or date of the first GAS procedure. Of note, the earliest index date for GAHT was in 2005 and earliest date for GAS was in 2013. For those who had not undergone medical therapy or surgical treatment, no index date was assigned.

Gender-affirming surgeries were categorized as the following: bottom (i.e. vaginoplasty, orchiectomy, phalloplasty, hysterectomy) or top (i.e. bilateral mastectomy, breast augmentation) as well as feminizing or masculinizing.

Sexual orientation and gender identity (SOGI) data is based on patient self-report, while legal sex (list here as EHR-reported sex) is based on government issued identification. This healthcare system launched the SOGI data questionnaire in 2018 and only recently has had initiatives to standardize and streamline collection of this data. Race and ethnicity were categorized based on

the options available in the electronic health record. Body mass index (BMI) was calculated in two ways: the first was a mean derived from the BMI before and closest to the index date and the second was the average of all BMIs prior to the index date. Patients' geographical information was also used and subset into rural, urban, and unknown based on zip code [33].

Statistical Methods

Descriptive statistics are reported as means (standard deviation) or frequencies (percentages). Chi-square and Student t-tests were used to compare the baseline demographics and comorbidities of the cohorts. Fisher's exact test was used when the Chi-square test was inadequate.

Data processing was performed using R (Version 3.6.3), RStudio (Version 1.2.5033), with appropriate packages. Statistical analysis was performed using R (Version 3.4.1), RStudio (Version 1.0.153). A two-sided test with a p-value of less than 0.05 was considered statistically significant. Analysis of the data collected as part of routine clinical care, and subsequent reporting of anonymized, aggregate data, was approved by the University of Utah Institutional Review Board (IRB_00159449).

Results

Based on the methods specified in Figure 1, the study initially identified 4,807 unique adults who met the ICD-9 or -10 diagnosis of gender dysphoria within this Utah-based health care system from 2003-2023 (Figure 1). After cohort verification with manual chart review, 43 adults were excluded for a final cohort size of 4,764 TGD adults. From 2003 – 2023, 1,216 TGD adults had undergone at least one GAS, 1,779 had actively received GAHT without undergoing GAS and

1,769 were not actively managed (had not undergone active medical therapy or surgical intervention).

Figure 1. Flow diagram of categorizing transgender and gender-diverse adults (2003-2023)

Table 4 compares the demographics of TGD adults who received GAHT and/or GAS versus those not actively managed. There was no significant difference in race or ethnicity between the two groups. Adults who underwent GAS and/or GAHT were more likely to have commercial insurance (75.0% vs 62.6%) and less likely to have Medicaid (10.3% vs 13.8%), Medicare (3.9% vs 9.7%) and unknown insurance (8.2% vs 11.3%) compared to adults who were not actively managed. Adults who underwent GAS and/or GAHT were more likely to be from Utah (87.2% vs 81.6%) and significantly higher percentage of people from urban areas (92.8% vs 89.5%).

Table 4. Demographics of transgender and gender-diverse adults undergoing gender-affirming surgery and/or gender-affirming hormone therapy versus not actively managed

	Not Actively Managed (N=1769)[a]	Surgery and/or hormone (N=2995)	All (N=4764)	p-value
**Race**				0.075
White or Caucasian	1367 (86.3%)	2545 (87.1%)	3912 (82.1%)	
Black or African American	35 (2.2%)	46 (1.6%)	81 (1.7%)	
Asian	20 (1.3%)	64 (2.2%)	84 (1.8%)	
American Indian/Alaska Native	33 (2.1%)	42 (1.4%)	75 (1.6%)	
Native Hawaiian/Pacific Islander	12 (0.8%)	19 (0.7%)	31 (0.7%)	
Other	117 (7.4%)	207 (7.1%)	324 (6.8%)	
Unknown race	185 (-)	72 (-)	257 (5.4%)	
**Ethnicity**				0.533
Hispanic/Latino	152 (9.9%)	301 (10.5%)	453 (9.5%)	
Not Hispanic/Latino	1391 (90.1%)	2566 (89.5%)	3957 (83.1%)	
Unknown ethnicity	226 (-)	128 (-)	354 (7.4%)	
**Marital status**				**<.001**
Married/Life partner	415 (26.4%)	612 (22.0%)	1027 (21.6%)	
Divorced/Legally separated	85 (5.4%)	133 (4.8%)	218 (4.6%)	

Widowed	32 (2.0%)	13 (0.5%)	45 (0.9%)	
Single	1042 (66.2%)	2022 (72.7%)	3064 (64.3%)	
Unknown/Other	195 (-)	215 (-)	410 (8.6%)	
**Sex (EHR reported)**				0.296
Female	963 (54.7%)	1576 (52.8%)	2539 (53.3%)	
Male	789 (44.8%)	1384 (46.4%)	2173 (45.6%)	
Nonbinary	9 (0.5%)	23 (0.8%)	32 (0.7%)	
Unknown sex	8 (-)	12 (-)	20 (0.4%)	
**Gender identity**				**<.001**
Female	220 (12.4%)	379 (12.7%)	599 (12.6%)	
Male	168 (9.5%)	266 (8.9%)	434 (9.1%)	
Transgender Female	220 (12.4%)	755 (25.2%)	975 (20.5%)	
Transgender Male	261 (14.8%)	685 (22.9%)	946 (19.9%)	
Non-binary	201 (11.4%)	336 (11.2%)	537 (11.3%)	
Unknown gender identity	699 (39.5%)	574 (19.2%)	1273 (26.7%)	
**Insurance status**				**<.001**
Commercial	1108 (62.6%)	2247 (75.0%)	3355 (70.4%)	
Medicaid	245 (13.8%)	309 (10.3%)	554 (11.6%)	
Medicare	171 (9.7%)	116 (3.9%)	287 (6.0%)	
Misc Government	45 (2.5%)	76 (2.5%)	121 (2.5%)	
Unknown insurance	200 (11.3%)	247 (8.2%)	447 (9.4%)	
**State**				**<.001**
Idaho	126 (7.1%)	113 (3.8%)	239 (5.0%)	
Nevada	44 (2.5%)	58 (1.9%)	102 (2.1%)	
Utah	1442 (81.6%)	2610 (87.2%)	4052 (85.1%)	
Wyoming	27 (1.5%)	42 (1.4%)	69 (1.4%)	
Other states[b]	129 (7.3%)	171 (5.7%)	300 (6.3%)	
Unknown state	1 (-)	1 (-)	2 (0.0%)	
**Rurality**				**<.001**
Urban	1583 (89.5%)	2777 (92.8%)	4360 (91.5%)	
Rural	185 (10.5%)	217 (7.2%)	402 (8.4%)	
Unknown	1 (-)	1 (-)	2 (0.0%)	

[a]Missing values were not included when calculating the p-values (chi-square test is used to compare categories, t-test is used to compare means)
[b]Other state include: AZ, CA, CO, CT, DC, FL, HI, IA, IL, IN, MA, KY, MD, MI, MN, MO, MS, NC, ND, NE, NH, MT, NM, NY, OH, OK, OR, PA, SC, SD, TN, TX, VA, WA

The demographics of TGD adults who received GAHT without GAS was also compared to those who had undergone GAS (Table 5). The mean age at first index date prescription was 26.8 years old for GAHT and 29.8 years old for GAS (p<0.001). There was no significant difference between the two groups in terms of race or ethnicity. The mean BMI was lower for the actively

managed GAHT group compared to the GAS group (27.6 vs 28.2, p=0.049). Adults who received GAHT without GAS were more likely to have commercial insurance (77.8% vs 71.0%) and Medicaid (12.3% vs 7.5%), and less likely to have Medicare (2.9% vs 5.2%) and unknown insurance (4.1% vs 14.3%) compared to the GAS group. Adults in the GAHT without GAS group were more likely to live in-state (95.3% vs 75.3%, p<0.001) and less likely to be from a rural area (6.1% vs 8.9%, p=0.005) compared to the GAS group.

Table 5. Demographics of active gender-affirming hormone therapy (GAHT) and gender-affirming surgery (GAS)

	**GAHT without GAS[a] (N=1779)**	**GAS (N=1216)**	**All (N=2995)**	**p-value**
**Age at first index date**				**<0.001**
<=20	454 (25.5%)	174 (14.3%)	628 (21.0%)	
21-30	923 (51.9%)	646 (53.1%)	1569 (52.4%)	
31-40	246 (13.8%)	237 (19.5%)	483 (16.1%)	
41-64	144 (8.1%)	137 (11.3%)	281 (9.4%)	
65+	12 (0.7%)	22 (1.8%)	34 (1.1%)	
**Age at first index date - Mean (SD)**	26.8 (9.26)	29.8 (10.9)	28.0 (10.1)	**<0.001**
**Therapy start year**				**<0.001**
2003-2016	141 (7.9%)	175 (14.4%)	316 (10.6%)	
2017-2018	179 (10.1%)	203 (16.7%)	382 (12.8%)	
2019	206 (11.6%)	137 (11.3%)	343 (11.5%)	
2020	270 (15.2%)	150 (12.3%)	420 (14.0%)	
2021	419 (23.6%)	210 (17.3%)	629 (21.0%)	
2022-2023	564 (31.7%)	341 (28.0%)	905 (30.2%)	
**Therapy start year – Mean (SD)**	2020 (2.49)	2020 (2.48)	2020 (2.50)	1.00
**Race**				0.372[b]
White or Caucasian	1516 (87.0%)	1029 (87.2%)	2545 (85.0%)	
Black or African American	25 (1.4%)	21 (1.8%)	46 (1.5%)	
Asian	36 (2.1%)	28 (2.4%)	64 (2.1%)	
American Indian/Alaska Native	22 (1.3%)	20 (1.7%)	42 (1.4%)	
Native Hawaiian/Pacific Islander	15 (0.9%)	4 (0.3%)	19 (0.6%)	
Other	129 (7.4%)	78 (6.6%)	207 (6.9%)	
Unknown race	36 (-)	36 (-)	72 (2.4%)	
**Ethnicity**				0.594
Hispanic/Latino	174 (10.2%)	127 (10.9%)	301 (10.1%)	
Not Hispanic/Latino	1529 (89.8%)	1037 (89.1%)	2566 (85.7%)	
Unknown ethnicity	76 (-)	52 (-)	128 (4.3%)	
**Body Mass Index (BMI) category[c]**				**<0.001**

Underweight (<18.5)	77 (5.5%)	26 (2.2%)	103 (3.4%)	
Normal (18.5 - 24.99)	561 (39.7%)	418 (35.0%)	979 (32.7%)	
Overweight (25.0 - 29.99)	344 (24.4%)	349 (29.2%)	693 (23.1%)	
Obese (>=30)	430 (30.5%)	402 (33.6%)	832 (27.8%)	
Unknown BMI	367 (-)	21 (-)	388 (13.0%)	
**Body Mass Index - Mean (SD)[c]**	27.6 (7.90)	28.2 (6.71)	27.9 (7.38)	**0.049**
**Body Mass Index - Mean (SD)[d]**	27.4 (7.69)	28.1 (6.66)	27.7 (7.24)	**0.018**
**Marital status**				**<0.001**
Married/Life partner	305 (18.6%)	307 (26.9%)	612 (20.4%)	
Divorced/Legally separated	76 (4.6%)	57 (5.0%)	133 (4.4%)	
Widowed	6 (0.4%)	7 (0.6%)	13 (0.4%)	
Single	1252 (76.4%)	770 (67.5%)	2022 (67.5%)	
Unknown/Other	140 (-)	75 (-)	215 (7.2%)	
**Sex (EHR[e] reported)**				**<0.001**
Female	782 (44.1%)	794 (65.6%)	1576 (52.6%)	
Male	980 (55.3%)	404 (33.4%)	1384 (46.2%)	
Nonbinary	10 (0.6%)	13 (1.1%)	23 (0.8%)	
Unknown sex	7 (-)	5 (-)	12 (0.4%)	
**Gender identity**				**<0.001**
Female	275 (15.5%)	104 (8.6%)	379 (12.7%)	
Male	146 (8.2%)	120 (9.9%)	266 (8.9%)	
Transgender Female	578 (32.5%)	177 (14.6%)	755 (25.2%)	
Transgender Male	340 (19.1%)	345 (28.4%)	685 (22.9%)	
Non-binary	194 (10.9%)	142 (11.7%)	336 (11.2%)	
Unknown gender identity	246 (13.8%)	328 (27.0%)	574 (19.2%)	
**Insurance status**				**<0.001**
Commercial	1384 (77.8%)	863 (71.0%)	2247 (75.0%)	
Medicaid	218 (12.3%)	91 (7.5%)	309 (10.3%)	
Medicare	53 (3.0%)	63 (5.2%)	116 (3.9%)	
Misc Government	51 (2.9%)	25 (2.1%)	76 (2.5%)	
Unknown insurance	73 (4.1%)	174 (14.3%)	247 (8.2%)	
**State**				**<0.001**
Idaho	17 (1.0%)	96 (7.9%)	113 (3.8%)	
Nevada	11 (0.6%)	47 (3.9%)	58 (1.9%)	
Utah	1695 (95.3%)	915 (75.3%)	2610 (87.1%)	
Wyoming	25 (1.4%)	17 (1.4%)	42 (1.4%)	
Other states[f]	31 (1.7%)	140 (11.5%)	171 (5.7%)	
Unknown state	0 (0%)	1 (-)	1 (0.0%)	
**Rurality**				**0.005**
Urban	1670 (93.9%)	1107 (91.1%)	2777 (92.7%)	
Rural	109 (6.1%)	108 (8.9%)	217 (7.2%)	
Unknown	0 (0%)	1 (-)	1 (0.0%)	

[a]Missing values were not included when calculating the p-values (chi-square test is used to compare categories, t-test is used to compare means)
[b]Fisher's exact test used
[c]Mean BMI before and closest to index date
[d]Mean BMI of all BMI's prior to index date
[e]EHR = Electronic Health Record
[f]Other state include: AZ, CA, CO, CT, HI, IL, KY, MD, MO, MS, MT, NM, NY, OH, OK, OR, PA, SC, SD, TN, TX, WA

Table 6 reports the descriptive characteristics for TGD adults actively managed (receiving 2 or more prescriptions) with either estrogen-based (n=1,325) or testosterone-based (n=1,075) GAHT. Compared to TGD adults on estrogen-based therapy, individuals on testosterone-based therapy were overall younger (mean age 29.3 vs 25.4 years old, p<0.001), more diverse (Hispanic/Latino: 7.2% vs 13.7%), and more likely to have a higher starting BMI (mean BMI at start of GAHT 26.8 vs 29.1, p<0.001). Adults on testosterone were more likely to have undergone gender-affirming surgery (35.8% vs 17.8%, p<0.001), specifically top surgery (29.2 vs 3.4%) compared to TGD adults on estrogen. For insurance status, those on estrogen-based GAHT were more likely to have Medicaid (12.5% vs 10.5%) and Medicare (4.7% vs 2.4%), and less likely to have commercial insurance (76.2% vs 81.3%) compared to those on testosterone-based GAHT.

Table 6. Demographics based on type of gender-affirming hormone therapy: Estrogen versus testosterone[a]

	**Estrogen (N=1325)**	**Testosterone (N=1075)**	**All (N=2400)**	**p-value[b]**
**Age at first hormone prescriptio**				**<0.001[c]**
18-20	235 (17.7%)	311 (28.9%)	546 (22.8%)	
21-30	670 (50.6%)	576 (53.6%)	1246 (51.9%)	
31-40	230 (17.4%)	139 (12.9%)	369 (15.4%)	
41-64	169 (12.8%)	46 (4.3%)	215 (9.0%)	
65+	21 (1.6%)	3 (0.3%)	24 (1.0%)	
**Age at first hormone prescription- Mean (SD)**	29.3 (11.1)	25.4 (7.54)	27.6 (9.84)	**<0.001**
**Therapy start year**				**0.029**
2005-2016	115 (8.7%)	136 (12.7%)	251 (10.5%)	
2017-2018	176 (13.3%)	134 (12.5%)	310 (12.9%)	
2019	171 (12.9%)	132 (12.3%)	303 (12.6%)	

2020	210 (15.8%)	167 (15.5%)	377 (15.7%)	
2021	314 (23.7%)	220 (20.5%)	534 (22.3%)	
2022-2023	339 (25.6%)	286 (26.6%)	625 (26.0%)	
**Therapy start year – Mean (SD)**	2020 (2.58)	2020 (2.57)	2020 (2.58)	0.188
**Race**				**0.004**
White or Caucasian	1153 (89.3%)	907 (85.6%)	2060 (85.8%)	
Black or African American	11 (0.9%)	25 (2.4%)	36 (1.5%)	
Asian	25 (1.9%)	22 (2.1%)	47 (2.0%)	
American Indian/ Alaska Native	19 (1.5%)	11 (1.0%)	30 (1.3%)	
Native Hawaiian/Pacific Islander	12 (0.9%)	7 (0.7%)	19 (0.8%)	
Other	71 (5.5%)	87 (8.2%)	158 (6.6%)	
Unknown race	34 (-)	16 (-)	50 (2.1%)	
**Ethnicity**				**<0.001**
Hispanic/Latino	91 (7.2%)	142 (13.7%)	233 (9.7%)	
Not Hispanic/Latino	1172 (92.8%)	896 (86.3%)	2068 (86.2%)	
Unknown ethnicity	62 (-)	37 (-)	99 (4.1%)	
**Body Mass Index category**[d]				**<0.001**
Underweight (<18.5)	53 (5.1%)	40 (4.5%)	93 (3.9%)	
Normal (18.5 - 24.99)	439 (42.6%)	294 (33.2%)	733 (30.5%)	
Overweight (25.0 - 29.99)	274 (26.6%)	220 (24.9%)	494 (20.6%)	
Obese (>=30)	265 (25.7%)	331 (37.4%)	596 (24.8%)	
Unknown BMI	294 (-)	190 (-)	484 (-)	
**Body Mass Index - Mean (SD)**[d]	26.8 (7.00)	29.1 (8.32)	27.8 (7.72)	**<0.001**
**Body Mass Index - Mean (SD)**[e]	26.6 (6.90)	28.7 (8.09)	27.6 (7.54)	**<0.001**
Married/Life partner	250 (20.4%)	232 (23.1%)	482 (20.1%)	
Divorced/Legally separated	85 (6.9%)	34 (3.4%)	119 (5.0%)	
Widowed	11 (0.9%)	1 (0.1%)	12 (0.5%)	
Single	878 (71.7%)	737 (73.4%)	1615 (67.3%)	
Unknown/Other	101 (-)	71 (-)	172 (7.2%)	
**Sex (EHR[f] reported)**				**<0.001**[c]
Female	392 (29.7%)	770 (71.6%)	1162 (48.4%)	
Male	921 (69.9%)	295 (27.4%)	1216 (50.7%)	
Nonbinary	5 (0.4%)	10 (0.9%)	15 (0.6%)	
Unknown sex	7 (-)	0 (-)	7 (0.3%)	
**Gender identity**				**<0.001**[§]
Female	336 (25.4%)	10 (0.9%)	346 (14.4%)	
Male	17 (1.3%)	206 (19.2%)	223 (9.3%)	
Transgender Female	721 (54.4%)	3 (0.3%)	724 (30.2%)	
Transgender Male	4 (0.3%)	534 (49.7%)	538 (22.4%)	
Non-binary	93 (7.0%)	161 (15.0%)	254 (10.6%)	
Unknown gender identity	154 (11.6%)	161 (15.0%)	315 (13.1%)	

**Insurance status**				**0.002**
Commercial	1010 (76.2%)	874 (81.3%)	1884 (78.5%)	
Medicaid	166 (12.5%)	113 (10.5%)	279 (11.6%)	
Medicare	62 (4.7%)	26 (2.4%)	88 (3.7%)	
Misc Government	40 (3.0%)	22 (2.0%)	62 (2.6%)	
Unknown insurance	47 (3.5%)	40 (3.7%)	87 (3.6%)	
**State**				0.386
Idaho	27 (2.0%)	15 (1.4%)	42 (1.8%)	
Nevada	12 (0.9%)	11 (1.0%)	23 (1.0%)	
Utah	1246 (94.0%)	1003 (93.3%)	2249 (93.7%)	
Wyoming	15 (1.1%)	17 (1.6%)	32 (1.3%)	
Other states[g]	25 (1.9%)	29 (2.7%)	54 (2.3%)	
**Gender-affirming surgery**				**<0.001**
No	1089 (82.2%)	690 (64.2%)	1779 (74.1%)	
Yes	236 (17.8%)	385 (35.8%)	621 (25.9%)	
**Gender-Affirming surgery type**				**<0.001**
Top	45 (3.4%)	314 (29.2%)	359 (15.0%)	
Bottom	134 (10.1%)	31 (2.9%)	165 (6.9%)	
Both	57 (4.3%)	40 (3.7%)	97 (4.0%)	
No surgery	1089 (82.2%)	690 (64.2%)	1779 (74.1%)	
**Rurality**				0.097
Urban	1253 (94.6%)	998 (92.8%)	2251 (93.8%)	
Rural	72 (5.4%)	77 (7.2%)	149 (6.2%)	

[a]Individuals had at least two unique prescriptions for hormone therapy
[b]Missing values were not included when calculating the p-values (chi-square test is used to compare categories, t-test is used to compare means)
[c]Fisher's exact test used
[d]Mean BMI before and closest to index date
[e]Mean BMI of all BMI's prior to index date
[f]EHR = Electronic Health Record
[g]Other state include: AZ, CA, CO, CT, HI, IL, KY, MD, MO, MS, MT, NM, NY, OH, OK, OR, PA, SC, SD, TN, TX, WA

Table 7 reports the descriptive characteristics for adults who underwent either feminizing or masculinizing gender-affirming surgery. The mean age of individuals who underwent feminizing surgery was significantly older than those who underwent masculinizing surgery (37.6 years old vs 27.3 years old, p<0.001). More than half (58.8%) of masculinizing surgeries were performed in adults between 21 and 30 years old. Although there was no significant difference in race

between surgery type, masculinizing surgery had a higher percentage of adults who identified as Hispanic/Latino (12.3% vs 6.8%) compared to adults who underwent feminizing surgery. Adults who underwent masculinizing surgery were more likely to have adults with unknown insurance status (18.1% vs 2.4%), more likely to have people from out-of-state (27.0% vs 17.3%), and more likely to have undergone top surgery (91.0% vs 24.8%) compared to those who underwent feminizing surgery. Figure 2 shows the temporal trends of individuals undergoing gender-affirming surgery since 2013. The number of GAS per year has almost doubled from 2019 to 2022.

Table 7. Demographics based on type of gender-affirming surgery: Feminizing versus Masculinizing

	**Feminizing (N=294)**	**Masculinizing (N=922)**	**All (N=1216)**	**p-value[a]**
**Age at index date[b]**				**<0.001**
<=20	11 (3.7%)	163 (17.7%)	174 (14.3%)	
21-30	104 (35.4%)	542 (58.8%)	646 (53.1%)	
31-40	87 (29.6%)	150 (16.3%)	237 (19.5%)	
41-64	72 (24.5%)	65 (7.0%)	137 (11.3%)	
65+	20 (6.8%)	2 (0.2%)	22 (1.8%)	
**Age at index date - Mean (SD)**	37.6 (14.1)	27.3 (8.16)	29.8 (10.9)	**<0.001**
**Surgery start year**				**<0.001**
2013-2016	8 (2.7%)	167 (18.1%)	175 (14.4%)	
2017-2018	24 (8.2%)	179 (19.4%)	203 (16.7%)	
2019	37 (12.6%)	100 (10.8%)	137 (11.3%)	
2020	42 (14.3%)	108 (11.7%)	150 (12.3%)	
2021	75 (25.5%)	135 (14.6%)	211 (17.3%)	
2022-2023	108 (36.6%)	233 (25.3%)	341 (28.0%)	
**Surgery start year – Mean (SD)**	2020 (1.71)	2020 (2.59)	2020 (2.48)	1.00
**Race**				0.087[c]
White or Caucasian	255 (89.2%)	774 (86.5%)	1029 (84.6%)	
Black or African American	1 (0.3%)	20 (2.2%)	21 (1.7%)	
Asian	8 (2.8%)	20 (2.2%)	28 (2.3%)	
American Indian/Alaska Native	7 (2.4%)	13 (1.5%)	20 (1.6%)	
Native Hawaiian/Pacific Islander	1 (0.3%)	3 (0.3%)	4 (0.3%)	
Other	13 (4.4%)	65 (7.3%)	78 (6.4%)	
Unknown race	9 (-)	27 (-)	36 (3.0%)	
**Ethnicity**				**0.009**

Hispanic/Latino	18 (6.8%)	109 (12.3%)	127 (10.4%)	
Not Hispanic/Latino	260 (93.2%)	777 (87.7%)	1037 (85.3%)	
Unknown ethnicity	16 (-)	36 (-)	52 (4.3%)	
**Body Mass Index category[d]**				**<0.001**
Underweight (<18.5)	12 (4.1%)	14 (1.6%)	26 (2.1%)	
Normal (18.5 - 24.99)	124 (42.3%)	294 (32.6%)	418 (34.4%)	
Overweight (25.0 - 29.99)	86 (29.4%)	263 (29.1%)	349 (28.7%)	
Obese (>=30)	70 (24.2%)	332 (36.8%)	402 (33.1%)	
Unknown BMI	2 (-)	19 (-)	21 (1.7%)	
**Body Mass Index - Mean (SD)[d]**	26.6 (6.12)	28.7 (6.81)	28.2 (6.71)	**<0.001**
**Body Mass Index - Mean (SD)[e]**	26.6 (6.08)	28.6 (6.77)	28.1 (6.66)	**<0.001**
**Marriage Status**				**<0.001[c]**
Married/Life partner	86 (31.4%)	221 (25.5%)	307 (25.2%)	
Divorced/Legally separated	33 (11.9%)	24 (2.8%)	57 (4.7%)	
Widowed	7 (2.5%)	0 (0%)	7 (0.6%)	
Single	150 (54.2%)	620 (71.7%)	770 (63.3%)	
Unknown/Other	18 (-)	57 (-)	75 (6.2%)	
**Sex (EHR[f] reported)**				0.090[c]
Female	191 (65.0%)	603 (65.7%)	794 (65.3%)	
Male	102 (35.5%)	302 (32.9%)	404 (33.2%)	
Nonbinary	0 (0%)	13 (1.4%)	13 (1.1%)	
Unknown sex	1 (-)	4 (-)	5 (0.4%)	
**Gender identity**				**<0.001[c]**
Female	86 (29.3%)	18 (2.0%)	104 (8.6%)	
Male	2 (0.7%)	118 (12.8%)	120 (9.9%)	
Transgender Female	174 (59.2%)	3 (0.3%)	177 (14.6%)	
Transgender Male	2 (0.7%)	343 (37.2%)	345 (28.4%)	
Non-binary	9 (3.1%)	133 (14.4%)	142 (11.7%)	
Unknown gender identity	21 (7.1%)	307 (33.3%)	328 (27.0%)	
**Insurance status**				**<0.001[c]**
Commercial	218 (74.1%)	645 (70.0%)	863 (71.0%)	
Medicaid	32 (10.9%)	59 (6.4%)	91 (7.5%)	
Medicare	35 (11.9%)	28 (3.0%)	63 (5.2%)	
Misc Government	2 (0.7%)	23 (2.5%)	25 (2.1%)	
Unknown insurance	7 (2.4%)	167 (18.1%)	174 (14.3%)	
**State**				**0.004[§]**
Idaho	19 (6.5%)	77 (8.4%)	96 (7.9%)	
Nevada	12 (4.1%)	35 (3.8%)	47 (3.9%)	
Utah	243 (82.7%)	672 (73.0%)	915 (75.2%)	
Wyoming	2 (0.7%)	15 (1.6%)	17 (1.4%)	
Other states[g]	18 (6.1%)	122 (13.2%)	140 (11.5%)	
Unknown state	0 (0%)	1 (-)	1 (0.1%)	

**Rurality**				0.931
Urban	267 (90.8%)	840 (91.2%)	1107 (91.0%)	
Rural	27 (9.2%)	81 (8.8%)	108 (8.9%)	
Unknown	0 (0%)	1 (-)	1 (0.1%)	
**Surgery type**				**<0.001**
Top	73 (24.8%)	839 (91.0%)	912 (75.0%)	
Bottom	157 (53.4%)	41 (4.4%)	198 (16.3%)	
Both	64 (21.8%)	42 (4.6%)	106 (8.7%)	

[a]Missing values were not included when calculating the p-values (chi-square test is used to compare categories, t-test is used to compare means)
[b]Index date = year of first surgery
[c] Fisher's exact test used
[d]Mean BMI before and closest to index date
[e]Mean BMI of all BMI's prior to index date
[f]EHR = Electronic Health Record
[g]Other state include: AZ, CA, CO, CT, HI, IL, KY, MD, MO, MS, MT, NM, NY, OH, OK, OR, PA, SC, SD, TN, TX, WA

Figure 2: Gender-affirming surgery trends at a Utah-based healthcare system (2013-2022)

Discussion

This paper outlines the steps utilized to describe adults who identify as TGD via electronic health record data with the goal to use this information as an a clinical and equity tool to answer important clinical questions that have largely remained unanswered because previous analyses of other cohorts were insufficiently powered. Almost fifteen percent (14.9%) of adults seeking gender-affirming care lived out of state, and 8.4% lived in rural areas. Almost two-thirds (65.3%) of the total volume of individuals who received GAHT and/or GAS at this healthcare institution had an index date between 2020 – May 2023. The exponential growth in patient volume is reflective of the growing needs of the community as well as the growing number of providers who can provide gender-affirming care within the institution particularly across state lines.

Almost three-quarters (73.4%) of TGD adults had an index date for gender-affirming care (GAHT or GAS) prior to the age of 30. Based on national surveys, researchers estimate the

percentage of teenagers and young adults who identify as TGD has doubled in the past five years, with 1.3% of 18- to 24-year old identifying as TGD [3]. In contrast, 0.5% of individuals 25 to 64 identify as TGD, and 0.3% of individuals 65 and older are TGD [3,20]. Thus, the number of individuals seeking out GAHT is expected to continue to grow. Furthermore, the number of GAS within this healthcare institution has nearly tripled from 2016 to 2019 [34]. Similar to the Wright et al's cohort study, when stratified by age, patients 19-30 years-old had the greatest number of procedures; top surgeries were the most common [34].

Insurance coverage for gender-affirming care, particularly GAS, is variable within the US [35]. In this cohort, 14.3% of all TGD adults who underwent GAS had unknown insurance status, and this increased to 18.1% for those who had masculinizing GAS. These individuals are assumed to be either uninsured or paid out of pocket for GAS services. Compared to the general US population, TGD adults are more likely to be uninsured, unemployed, and living in poverty [36–38]. Even for those with insurance, they commonly face insurance denials for gender-affirming treatments as they are often considered elective cosmetic procedures [35]. Although restrictions for gender-affirming services have been receding, the co-pay can still be cost prohibitive [37]. Further research is needed to explore the differences amongst those with unknown insurance status – who has the finances to pursue gender-affirming care compared to those who cannot access care due to insurance barriers.

Nationally, roughly one in six (16%) TGD individuals live in rural areas [3,39]. This Utah-based cohort data categorized 8.4% of TGD adults living in rural areas, and this percentage increased to 10.5% for adults who met the criteria for TGD but had not sought active GAHT or GAS. The discrepancy in the percentage of adults living in rural areas and percentage of those seeking gender-affirming care is attributed to significant health disparities, one of which includes access

to healthcare [40–42]. The rapid adoption of telehealth during the COVID-19 pandemic bolstered the total number of TGD visits, for both new and established TGD adults [43]. Increase in access amongst those in rural communities is not as clear. Transgender and gender-diverse adults living in rural areas are twice as likely as their cisgender peers to be uninsured, and rural TGD people of color are three times as likely as their White, cisgender neighbors to be uninsured [41]. Further research is needed to examine how telehealth has affected access to gender-affirming care in rural communities, and how insurance status affects access.

Research studies focused on answering clinical questions related to gender-affirming hormone therapy have been exponentially growing. Although a national longitudinal cohort study is actively trending the physical, mental and social health data of TGD adults, the results of their study will not be available for years [44]. This delay in evaluating gender-affirming services presents a major barrier for the providers and patients facilitating and receiving care now. Currently, healthcare providers rely on guidelines published by WPATH [1], University of California San Francisco [45], Fenway [46], or the Endocrine Society [47]. These consensus-based guidelines have relied on findings from smaller cohort studies, but the number of larger scale studies is growing. Additionally, studies comparing health outcomes between those who underwent gender-affirming care (surgical or medical therapy) versus not is also growing [34,48,49]. The goal of creating this population cohort is to start answering clinically pertinent questions about patient outcomes as well as identifying health inequities.

A strength and unique aspect of this study is each person was assigned an index date based on their initial gender-affirming hormone therapy prescription or date of first gender-affirming surgery. This will allow future studies to track trends over time including laboratory values, medication dosages, and the incidence and prevalence of certain health conditions. Although, the

demographics of this mountain west healthcare system is not a nationally representative sample, it is reflective of the state's demographics. Many studies looking at the population of TGD adults have been concentrated along both coasts, and this represents the first cohort of its kind in a mountain west, conservative state.

Limitations

Reliance on ICD-9 and ICD-10 codes for gender dysphoria places undue emphasis on medical diagnoses billed by healthcare providers. The number of TGD adults estimated here is likely an underestimate. Some individuals who experience gender dysphoria or gender incongruence may choose not to disclose their concerns to healthcare professionals due to mistrust of the healthcare system [50–52]. Before insurance coverage changes, certain TGD adults explicitly requested that billing codes related to gender dysphoria not be used, as their insurance companies refused to cover these medical services. A few TGD adults who still had parental health insurance also requested different codes because they hadn't disclosed their gender identity yet. Consequently, providers used alternative codes such as endocrine disorder, unspecified. Furthermore, there are individuals who do not identify as TGD but may have been captured in this cohort due to coding error (such as, but limited to, those captured on manual chart review). The index date assigned is based on the date of the individual's first prescription for estrogen or testosterone within this mountain west healthcare system. This data cannot differentiate if this is the individual's first prescription to start gender-affirming hormone therapy or if the individual had already been on GAHT and was transferring their care to this healthcare system.

Conclusions

In conclusion, the findings from this dataset underscore a significant alignment with national trends indicating a rapid increase in the number of TGD adults actively pursuing gender-affirming care. What distinguishes this dataset is the creation of an index date to trend changes both from gender-affirming hormone therapy as well as gender-affirming surgeries. The goal is to utilize this as a clinical and health equity tool further advance the quality of health care provided to the TGD community. There is a critical need for targeted interventions and policy initiatives to bridge the healthcare divide, ensuring equitable access to life-affirming treatments for all, regardless of people’s geographic location.

Acknowledgements: University of Utah Department of Family and Preventive Medicine Health Studies Fund Clinician Scholars Program with support from Research Manager Eliza Taylor, MPH

References

1. Coleman E, Radix AE, Bouman WP, Brown GR, de Vries ALC, Deutsch MB, et al. Standards of Care for the Health of Transgender and Gender Diverse People, Version 8. Int J Transgender Health. 23(Suppl 1):S1–259.

2. Baker KE. Findings From the Behavioral Risk Factor Surveillance System on Health-Related Quality of Life Among US Transgender Adults, 2014-2017. JAMA Intern Med. 2019 Aug;179(8):1141–4.

3. Herman, J.L., Flores, A.R., O'Neill, K.K L|. How Many Adults and Youth Identify as Transgender in the United States? [Internet]. The Williams Institute, UCLA School of Law; [cited 2023 Nov 13]. Available from: https://williamsinstitute.law.ucla.edu/publications/trans-adults-united-states/

4. Meerwijk EL, Sevelius JM. Transgender Population Size in the United States: a Meta-Regression of Population-Based Probability Samples. Am J Public Health. 2017 Feb;107(2):e1–8.

5. Patterson CJ, Sepúlveda MJ, White J, editors. Understanding the Well-Being of LGBTQI+ Populations [Internet]. Washington, D.C.: National Academies Press; 2020 [cited 2023 Apr 26]. Available from: https://www.nap.edu/catalog/25877

6. Radix AE, Lelutiu-Weinberger C, Gamarel KE. Satisfaction and Healthcare Utilization of Transgender and Gender Non-Conforming Individuals in NYC: A Community-Based Participatory Study. LGBT Health. 2014 Dec;1(4):302–8.

7. Roberts TK, Fantz CR. Barriers to quality health care for the transgender population. Clin Biochem. 2014 Jul;47(10–11):983–7.

8. Turban JL, Beckwith N, Reisner SL, Keuroghlian AS. Association Between Recalled Exposure to Gender Identity Conversion Efforts and Psychological Distress and Suicide Attempts Among Transgender Adults. JAMA Psychiatry. 2020 Jan 1;77(1):68–76.

9. Reisner SL, White Hughto JM, Gamarel KE, Keuroghlian AS, Mizock L, Pachankis J. Discriminatory experiences associated with posttraumatic stress disorder symptoms among transgender adults. J Couns Psychol. 2016 Oct;63(5):509–19.

10. Seelman KL, Colón-Diaz MJP, LeCroix RH, Xavier-Brier M, Kattari L. Transgender Noninclusive Healthcare and Delaying Care Because of Fear: Connections to General Health and Mental Health Among Transgender Adults. Transgender Health. 2017 Dec;2(1):17–28.

11. Hughto JMW, Quinn EK, Dunbar MS, Rose AJ, Shireman TI, Jasuja GK. Prevalence and Co-occurrence of Alcohol, Nicotine, and Other Substance Use Disorder Diagnoses Among US Transgender and Cisgender Adults. JAMA Netw Open. 2021 Feb 4;4(2):e2036512.

12. Rowe C, Santos GM, McFarland W, Wilson EC. Prevalence and correlates of substance use among trans*female youth ages 16–24 years in the San Francisco Bay Area. Drug Alcohol Depend. 2015 Feb 1;147:160–6.

13. Becasen JS, Denard CL, Mullins MM, Higa DH, Sipe TA. Estimating the Prevalence of HIV and Sexual Behaviors Among the US Transgender Population: A Systematic Review and Meta-Analysis, 2006–2017. Am J Public Health. 2019 Jan;109(1):e1–8.

14. Baral SD, Poteat T, Strömdahl S, Wirtz AL, Guadamuz TE, Beyrer C. Worldwide burden of HIV in transgender women: a systematic review and meta-analysis. Lancet Infect Dis. 2013 Mar 1;13(3):214–22.

15. Reisner SL, Murchison GR. A global research synthesis of HIV and STI biobehavioral risks in female-to-male (FTM) transgender adults. Glob Public Health. 2016;11(7–8):866–87.

16. White Hughto JM, Reisner SL, Pachankis JE. Transgender stigma and health: A critical review of stigma determinants, mechanisms, and interventions. Soc Sci Med. 2015 Dec 1;147:222–31.

17. Hendricks ML, Testa RJ. A conceptual framework for clinical work with transgender and gender nonconforming clients: An adaptation of the Minority Stress Model. Prof Psychol Res Pract. 2012;43(5):460–7.

18. Valentine SE, Shipherd JC. A systematic review of social stress and mental health among transgender and gender non-conforming people in the United States. Clin Psychol Rev. 2018 Dec;66:24–38.

19. Sanchez NF, Sanchez JP, Danoff A. Health Care Utilization, Barriers to Care, and Hormone Usage Among Male-to-Female Transgender Persons in New York City. Am J Public Health. 2009 Apr;99(4):713–9.

20. Herman JL, Flores AR, Brown TN, Wilson BD, Conron KJ. Age of individuals who identify as transgender in the United States. eScholarship, University of California; 2017.

21. Christian R, Mellies AA, Bui AG, Lee R, Kattari L, Gray C. Measuring the Health of an Invisible Population: Lessons from the Colorado Transgender Health Survey. J Gen Intern Med. 2018 Oct;33(10):1654–60.

22. Streed CG, McCarthy EP, Haas JS. Self-Reported Physical and Mental Health of Gender Nonconforming Transgender Adults in the United States. LGBT Health. 2018 Oct 1;5(7):443–8.

23. Feldman JL, Luhur WE, Herman JL, Poteat T, Meyer IH. Health and Health Care Access in the U.S. Transgender Population Health (TransPop) Survey. Andrology. 2021 Nov;9(6):1707–18.

24. Lett E, Asabor EN, Beltrán S, Dowshen N. Characterizing Health Inequities for the U.S. Transgender Hispanic Population Using the Behavioral Risk Factor Surveillance System. Transgender Health. 2021 Oct 4;6(5):275–83.

25. Jasuja GK, de Groot A, Quinn EK, Ameli O, Hughto JMW, Dunbar M, et al. Beyond Gender Identity Disorder Diagnosis Codes: An Examination of Additional Methods to Identify Transgender Individuals in Administrative Databases. Med Care. 2020 Oct;58(10):903–11.

26. Wolfe HL, Reisman JI, Yoon SS, Blosnich JR, Shipherd JC, Vimalananda VG, et al. Validating Data-Driven Methods for Identifying Transgender Individuals in the Veterans Health Administration of the US Department of Veterans Affairs. Am J Epidemiol. 2021 Sep 1;190(9):1928–34.

27. Dubin S, Cook T, Liss A, Doty G, Moore K, Greene R, et al. Comparing Electronic Health Record Domains' Utility to Identify Transgender Patients. Transgender Health. 2022 Feb 14;7(1):78–84.

28. Roblin D, Barzilay J, Tolsma D, Robinson B, Schild L, Cromwell L, et al. A novel method for estimating transgender status using electronic medical records. Ann Epidemiol. 2016 Mar;26(3):198–203.

29. Brown GR, Jones KT. Racial Health Disparities in a Cohort of 5,135 Transgender Veterans. J Racial Ethn Health Disparities. 2014 Dec 1;1(4):257–66.

30. Quinn VP, Nash R, Hunkeler E, Contreras R, Cromwell L, Becerra-Culqui TA, et al. Cohort profile: Study of Transition, Outcomes and Gender (STRONG) to assess health status of transgender people. BMJ Open. 2017 Dec 27;7(12):e018121.

31. Wright MA, Doll KM, Myers E, Carpenter WR, Gartner DR, Robinson WR. Changing trends in Black-White racial differences in surgical menopause: a population-based study. Am J Obstet Gynecol. 2021 Nov 1;225(5):502.e1-502.e13.

32. Sun M, Choueiri TK, Hamnvik OPR, Preston MA, De Velasco G, Jiang W, et al. Comparison of Gonadotropin-Releasing Hormone Agonists and Orchiectomy: Effects of Androgen-Deprivation Therapy. JAMA Oncol. 2016 Apr 1;2(4):500–7.

33. Hall SA, Kaufman JS, Ricketts TC. Defining Urban and Rural Areas in U.S. Epidemiologic Studies. J Urban Health. 2006 Mar;83(2):162–75.

34. Wright JD, Chen L, Suzuki Y, Matsuo K, Hershman DL. National Estimates of Gender-Affirming Surgery in the US. JAMA Netw Open. 2023 Aug 23;6(8):e2330348.

35. Padula WV, Baker K. Coverage for Gender-Affirming Care: Making Health Insurance Work for Transgender Americans. LGBT Health. 2017 Aug;4(4):244–7.

36. GONZALES G, HENNING- SMITH C. Barriers to Care Among Transgender and Gender Nonconforming Adults. Milbank Q. 2017 Dec;95(4):726–48.

37. Baker K, Restar A. Utilization and Costs of Gender-Affirming Care in a Commercially Insured Transgender Population. J Law Med Ethics. 50(3):456–70.

38. Patel H, Camacho JM, Salehi N, Garakani R, Friedman L, Reid CM. Journeying Through the Hurdles of Gender-Affirming Care Insurance: A Literature Analysis. Cureus. 15(3):e36849.

39. Crissman HP, Berger MB, Graham LF, Dalton VK. Transgender Demographics: A Household Probability Sample of US Adults, 2014. Am J Public Health. 2017 Feb;107(2):213–5.

40. Jackson KJ, Tomlinson S. A review of top performing rural community and critical access hospitals' web resources for transgender patients in the United States. Sex Reprod Healthc Off J Swed Assoc Midwives. 2021 Sep;29:100627.

41. Movement Advancement Project. Where We Call Home: Transgender People in Rural America [Internet]. 2019 [cited 2023 Sep 19]. Available from: https://www.lgbtmap.org/file/Rural-Trans-Report-Nov2019.pdf

42. Renner J, Blaszcyk W, Täuber L, Dekker A, Briken P, Nieder TO. Barriers to Accessing Health Care in Rural Regions by Transgender, Non-Binary, and Gender Diverse People: A Case-Based Scoping Review. Front Endocrinol [Internet]. 2021 [cited 2023 Apr 4];12. Available from: https://www.frontiersin.org/articles/10.3389/fendo.2021.717821

43. Lock L, Anderson B, Hill BJ. Transgender Care and the COVID-19 Pandemic: Exploring the Initiation and Continuation of Transgender Care In-Person and Through Telehealth. Transgender Health. 2022 Apr 11;7(2):165–9.

44. Lunn MR, Lubensky M, Hunt C, Flentje A, Capriotti MR, Sooksaman C, et al. A digital health research platform for community engagement, recruitment, and retention of sexual and gender minority adults in a national longitudinal cohort study——The PRIDE Study. J Am Med Inform Assoc JAMIA. 2019 Jun 4;26(8–9):737–48.

45. Deutsch MB. Guidelines for the Primary and Gender-Affirming Care of Transgender and Gender Nonbinary People [Internet]. 2nd ed. UCSF Gender Affirming Health Program, Department of Family and Community Medicine, University of California San Francisco; 2016. Available from: https://transcare.ucsf.edu/guidelines

46. Thompson J, Hopwood R, deNormand S, Cavanaugh T. Medical Care of Trans and Gender Diverse Adults. Boston: Fenway Health; 2021 Mar.

47. Hembree WC, Cohen-Kettenis PT, Gooren L, Hannema SE, Meyer WJ, Murad MH, et al. Endocrine Treatment of Gender-Dysphoric/Gender-Incongruent Persons: An Endocrine Society Clinical Practice Guideline. J Clin Endocrinol Metab. 2017 Nov 1;102(11):3869–903.

48. Oles N, Darrach H, Landford W, Garza M, Twose C, Park CS, et al. Gender Affirming Surgery: A Comprehensive, Systematic Review of All Peer-reviewed Literature and

Methods of Assessing Patient-centered Outcomes (Part 1: Breast/Chest, Face, and Voice). Ann Surg. 2022 Jan 1;275(1):e52–66.

49. Oles N, Darrach H, Landford W, Garza M, Twose C, Park CS, et al. Gender Affirming Surgery: A Comprehensive, Systematic Review of All Peer-reviewed Literature and Methods of Assessing Patient-centered Outcomes (Part 2: Genital Reconstruction). Ann Surg. 2022 Jan 1;275(1):e67–74.

50. James SE, Herman JL, Rankin S, Keisling M, Mottet L, Anafi M. The report of the 2015 US transgender survey. Washington, D.C: National Center for Transgender Equality; 2016.

51. Kattari SK, Bakko M, Langenderfer-Magruder L, Holloway BT. Transgender and Nonbinary Experiences of Victimization in Health care. J Interpers Violence. 2021 Dec 1;36(23–24):NP13054–76.

52. Clark KD, Luong S, Lunn MR, Flowers E, Bahalkeh E, Lubensky ME, et al. Healthcare Mistreatment, State-Level Policy Protections, and Healthcare Avoidance Among Gender Minority People. Sex Res Soc Policy. 2022 Dec 1;19(4):1717–30.

S1 Table. List of estrogen- and testosterone- based medications included

Click here to access/download;Figure;Figure 1_PLoS.docx

Figure 1. Flow diagram of categorizing transgender and gender diverse adults (2003-2023)

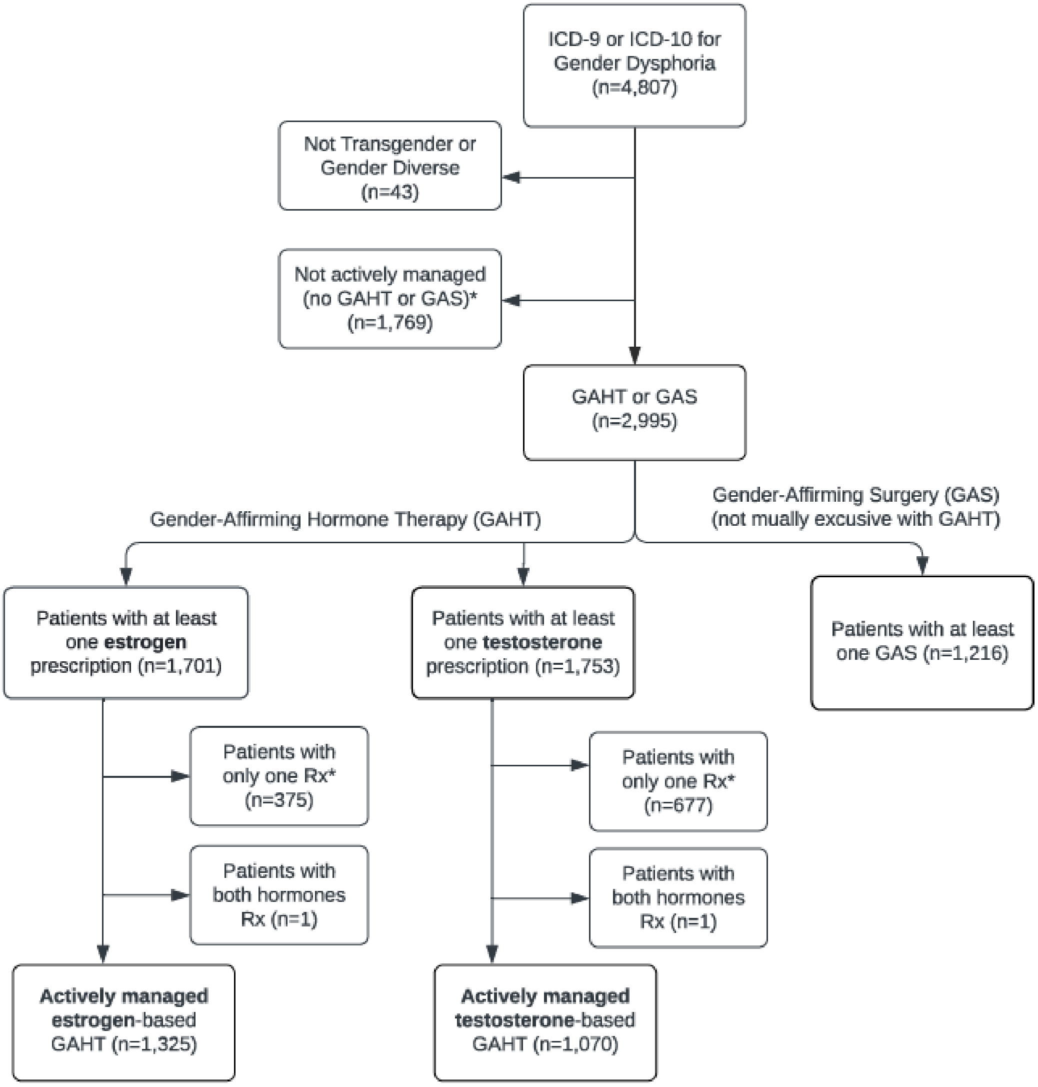

*Patients with only one prescription (Rx) were excluded from the actively managed GAHT group and counted in the "Not actively managed group"

Click here to access/download;Figure;Figure 2_PLOS.docx

Figure 2: Gender-affirming surgery trends at a Utah-based healthcare system (2013-2022)

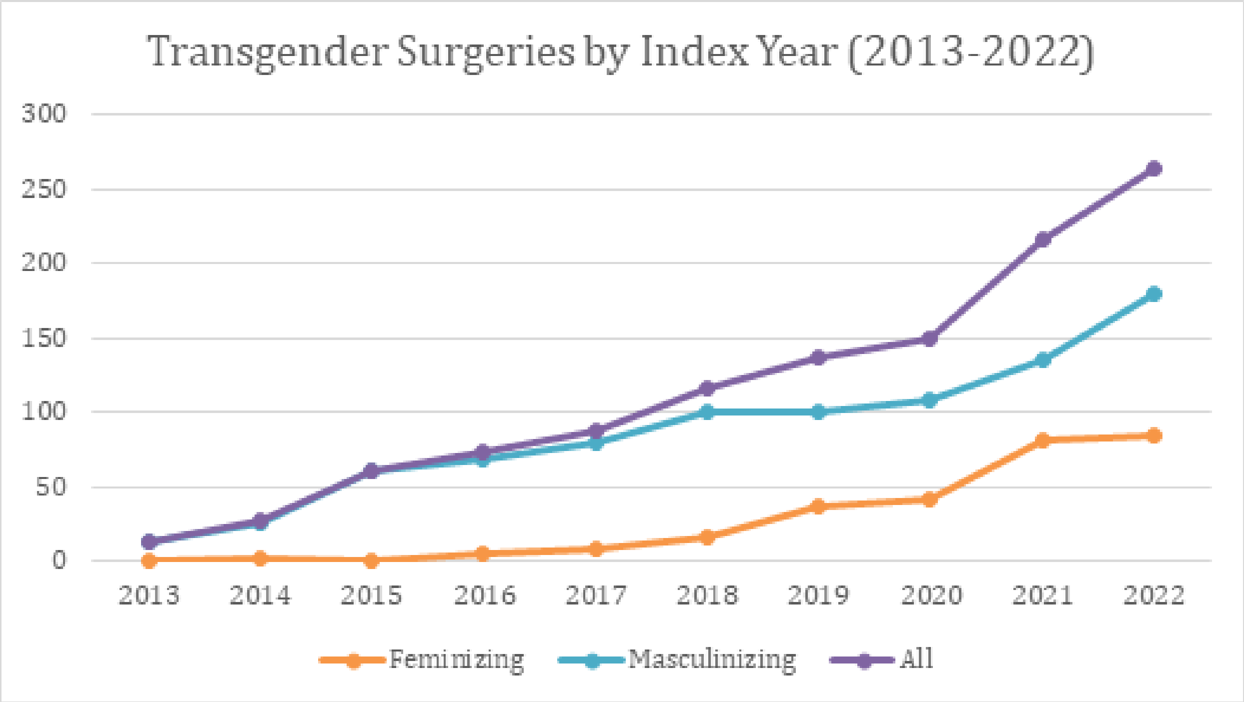

S1 Table. List of estrogen- and testosterone- based medications included

Click here to access/download
Supporting Information
S1 Table_PLOS.docx
