# Supplementary material for: Characteristics of a transgender and gender-diverse patient population in Utah: Use of electronic health records to advance clinical and health equity research
Source: PLoS One. 2024 May 7;19(5):e0302895. doi: 10.1371/journal.pone.0302895 (PMC11075904; doi:10.1371/journal.pone.0302895)
Supplement: Attachment — Submitted filename: PONE-D-23-40640 Review.docx [file pone.0302895.s003.docx]

**Characteristics of a transgender and gender-diverse patient population in Utah: Use of**

**electronic health records to advance clinical and health equity research**

I would like to thank the authors for the opportunity to review their manuscript. This study provides a blueprint for how to identify and describe TGD adults and their usage of gender-affirming care within a health care system based on ICD codes. While I do recommend revisions, my hope is that this article reaches publication given its importance.

**Overall**

I am curious about the decision to include BMI. I understand that many surgeons have BMI requirements for accessing surgeries, but it seemed like an add-on. There was no mention of BMI/weight in the lit review or the discussion, making me question its importance to your study. Research has shown the BMI to be an outdated measure, and further research has shown that the use of the BMI can be used to (1) gatekeep surgeries, and (2) contribute to weight stigma, particularly for folks in larger/fat bodies.

**Abstract**

Delete the word “have” in this sentence: “During this time frame, 2,995 adults have received gender-affirming hormone therapy (GAHT)…”

**Introduction**

Line 80: I recommend choosing a different word than “TGD experience”

The introduction/background is informative and grounds the reader for what’s coming next.

**Methods**

Line 117: “…and 22 were excluded as they did not meet the criteria for TGD.” This sentence confused me because it left me wondering what the criteria is for TGD. In line 119, you write something similar, except in parenthesis you include, “(all postmenopausal cis women on hormone replacement therapy).” Will you clarify prior to line 117 what the criteria are for TGD in this study? And if criteria aren’t met, will you specify why that is like you did in line 119? I’m also wondering if this could be an issue with phrasing (“criteria for TGD”).

Lines 112-125: I recommend re-writing this paragraph in a way that is more accessible to the reader. I found myself a bit lost reading this paragraph, particularly around the cohort being TGD-identified with 100% accuracy and then some folks not meeting criteria for TGD. In order for these methods to be replicated, I think there needs to be a bit more clarity.

I appreciate the way your team used the specific hormone therapy as categories to be more inclusive of nonbinary and TGD folks.

The statistical methods section makes sense and flows well.

**Results**

In your tables, I recommend removing the word Caucasian since it’s outdated.

**Discussion**

Line 267: delete “an”

In the discussion, I think it could be worthwhile to note that although there are more known trans people, trans people have always existed and may not have felt comfortable/safe being as visible as they are today. Access to care is becoming more widespread and known, making it more likely for trans folks to engage with the health care system to access gender-affirming care. I think it’s important for readers to understand that there isn’t necessarily an increase in trans people, but there is an increase in trans visibility and trans people accessing care.

**Conclusion**

Lines 344-346: “In conclusion, the findings from this dataset underscore a significant alignment with national trends indicating a rapid increase in the number of TGD adults actively pursuing gender-affirming care.” I recommend re-writing this sentence. Unintentionally, by using language such as “rapid increase,” this may fuel arguments around social contagion and push the narrative that the number of TGD people is increasing rapidly, and the number of folks seeking gender-affirming care is rapidly increasing. It is clear that this is not your intention, but I immediately had this thought when reading this.

The closing sentence of the conclusion is great!

Again, thank you for the opportunity to review your manuscript. I can tell how intentional this work was conducted, and the thoughtfulness of the research team. I am recommending revisions to increase clarity and accessibility for readers.
