# Supplementary material for: Characteristics of a transgender and gender-diverse patient population in Utah: Use of electronic health records to advance clinical and health equity research
Source: PLoS One. 2024 May 7;19(5):e0302895. doi: 10.1371/journal.pone.0302895 (PMC11075904; doi:10.1371/journal.pone.0302895)
Supplement: Attachment — Submitted filename: Response to Reviewers.docx [file pone.0302895.s004.docx]

**Response to Reviewers**

Dear Daniel Antwi-Amoabeng, MD. MSc

Thank you for reviewing our manuscript titled *“*Characteristics of a transgender and gender-diverse patient population in Utah: Use of electronic health records to advance clinical and health equity research*”* (Manuscript ID PONE-D-23-40640). We appreciate your comments and the reviewer’s constructive feedback. We have addressed all comments and revised the manuscript accordingly. Enclosed is a point-by-point response.

Tiffany F. Ho, MD, MPH, FAAFP

Assistant Professor, Clinical

Department of Family and Preventative Medicine

University of Utah School of Medicine

375 Chipeta Way, Suite A

Salt Lake City, UT 84108

[Tiffany.ho@hsc.utah.edu](mailto:Tiffany.ho@hsc.utah.edu)

| **Reviewer Comments** | **How/where addressed** |
| --- | --- |
| *Academic Editor* |  |
| 1.1 Please ensure that your manuscript meets PLOS ONE's style requirements, including those for file naming. | We have formatted our manuscript to meet PLOS ONES’s Requirements. |
| 1.2 We note that the grant information you provided in the ‘Funding Information’ and ‘Financial Disclosure’ sections do not match.  When you resubmit, please ensure that you provide the correct grant numbers for the awards you received for your study in the ‘Funding Information’ section. | Unable to find financial disclosures section. I had emailed PLOS One and never received a reply. I’ve confirmed that the funding information is correct. |
| 1.3 When completing the data availability statement of the submission form, you indicated that you will make your data available on acceptance. We strongly recommend all authors decide on a data sharing plan before acceptance, as the process can be lengthy and hold up publication timelines. | We have uploaded our data and made it available to access. |
| 1.4 Please include your full ethics statement in the ‘Methods’ section of your manuscript file. In your statement, please include the full name of the IRB or ethics committee who approved or waived your study, as well as whether or not you obtained informed written or verbal consent. If consent was waived for your study, please include this information in your statement as well | This has been updated in the methods section. |
| 1.5  Please include captions for your Supporting Information files at the end of your manuscript, and update any in-text citations to match accordingly. | This has been updated |
| 1.6 Please review your reference list to ensure that it is complete and correct. If you have cited papers that have been retracted, please include the rationale for doing so in the manuscript text, or remove these references and replace them with relevant current references. | This has been reviewed |

| **Reviewer Comments** | **How/where addressed** |
| --- | --- |
| Reviewer #1 |  |
| 2.1 Please can the authors describe in the methods to what degree have TGD community been involved in the design and conduct of the research, and how will they be involved in future cohort analysis and dissemination of results. | Thank you for this comment, we have added a paragraph in the methods section detailed the involvement of the TGD community. |
| 2.2 The different gender affirming surgeries have quite different rates of uptake which also vary within healthcare systems. This granularity is seldom published and would be valuable not only to scientists and physicians, but to policy makers. For this reason, describing the frequency of GAS in smaller categories e.g. hysterectomy BSO vs phallo/metoidio vs chest would be helpful. | Thank you for this feedback, we agree the level of granularity is valuable and originally planned to submit a separate manuscript on it. We have included a table that lists the frequencies of each surgery. |
| 2.3 Introduction line 69-74 It may be worth commenting that there is geographical variation in the incidence prevalence of some of these conditions which may reflect the factors below. | We recognize the audience is not limited to the United States, and this comment is likely referring to countries with widespread support do not have these health disparities. We have edited our introduction. |
| 2.4 Methods line 96-98 Why not use ICD-11 given that the end date for the cohort was April 2023? Is this not used there. If not being used, this should be justified. | ICD-11 codes are currently no used in the United States. |
| 2.5 Methods line 120 This is not the preferred term - please use "gender corresponding the the sex they were assigned at birth" or similar language. | Thank you for this clarification. Phrase changed to “four adults had detransitioned to a gender corresponding with their sex assigned at birth” |
| 2.6 Methods line 124 This is a good point - was there a way of verifying that GAS were actually done with the intent of being gender affirming? | Thank for this comment. In addition to the manual chart review of the 93 adults who had surgery types that were incongruent with each other or with the category of GAHT, another 30 patients were randomly reviewed to confirm the indication for surgery was indeed for gender-affirming care. This detail has been added to the manuscript. |
| 2.7 Methods line 149 There is quite a difference in terms of invasiveness of hysterectomy vs the other surgeries here, and hence i wonder if worth separating for purposes of analysis. Were bilateral salpingoophorectomy also included? This has relevance for thinking about future gynaecologica | We agree that there is variable invasiveness of the procedures listed. Our long-term goal is to assess differences in surgical approach based on patient characteristics. The goals of this study were to capture all potential procedures in broad terms. We also clarified the description of the category, as salpingo-oophorectomy was also included. |
| 2.8 Results line 219-221 But is age a confounder here? Please comment | We agree age is likely a counfounder. This paper intended to provide descriptive analysis of the variables. In our next paper, we intend to perform multi-variate analyses to see how factors such as age, race/ethnicity, BMI, rurality impacts the rates of GAHT and GAS. |
| 2.9 line 250 As mentioned, splitting by surgery types is very relevant here so that patterns of hysterectomy are drawn out (given relevance to gynaecological care), other gynae surgeries, and also top surgery in trans women. These also have implications for international providers thinking about which surgeries should be funded. | Thank you for this comment. We agree that this is very relevant for long-term care and management in this population. However, in our current system, the practical determination of which codes align with which procedures is more difficult in administrative review. Specifically, we believe that the general codes are correct in identifying organ removal vs. No organ removal, but the specific patterns of hysterectomy are not reliably coded in our dataset. This will likely be a topic for a future publication with extensive manual review. |
| 2.10 Discussion line 272-274 Also increased rates of disclosure related to cultural change. This should be added. | Thank you for this recommendation. We have added references and text about how younger individuals may have lower concerns with or more motivation for disclosure. Also younger individuals report feeling safer coming out than in previous studies. |
| 2.11 Discussion line 276-278 Again please comment on disclosure and factors why | Added and referenced how community infrastructure and tools like social media to support queer youth |
| 2.12 Discussion line 328 Please comment on lack of ICD-11 | ICD-11 is not currently being used in the United States |
|  |  |
| *Reviewer #2* |  |
| 3.1 decision to include BMI | Thank you for this point of clarification, we included surgeons often use BMI to determine surgical candidacy. |
| 3.2 Abstract – delete the word ‘have’ | Change made |
| 3.3 Introduction - Line 80: I recommend choosing a different word than “TGD experience” | Changed to phrase “TGD individuals pursuing transition-related options” to underscore reason for visibility, and the narrower focus of medical care |
| 3.4 Methods - Line 117: “…and 22 were excluded as they did not meet the criteria for TGD.” This sentence confused me because it left me wondering what the criteria is for TGD. In line 119, you write something similar, except in parenthesis you include, “(all postmenopausal cis women on hormone replacement therapy).” Will you clarify prior to line 117 what the criteria are for TGD in this study? And if criteria aren’t met, will you specify why that is like you did in line 119? I’m also wondering if this could be an issue with phrasing (“criteria for TGD”). | Thank for this point of clarification. We have edited ‘criteria for TGD’ to specifically state the individual’s gender identity was congruent with their sex assigned at birth. |
| 3.5 Methods - Lines 112-125: I recommend re-writing this paragraph in a way that is more accessible to the reader. I found myself a bit lost reading this paragraph, particularly around the cohort being TGD-identified with 100% accuracy and then some folks not meeting criteria for TGD. In order for these methods to be replicated, I think there needs to be a bit more clarity. | Thank you for the feedback, this paragraph has been organized for better clarity. |
| 3.6 Results - In your tables, I recommend removing the word Caucasian since it’s outdated | Removed |
| 3.7 Discussion - Line 267: delete “an” | Deleted |
| 3.8 In the discussion, I think it could be worthwhile to note that although there are more known trans people, trans people have always existed and may not have felt comfortable/safe being as visible as they are today. Access to care is becoming more widespread and known, making it more likely for trans folks to engage with the health care system to access gender-affirming care. I think it’s important for readers to understand that there isn’t necessarily an increase in trans people, but there is an increase in trans visibility and trans people accessing care. | This is definitely related to concerns about supporting ROGD as a hypothesis. I think there’s a historical element of TGD medical care we can tie in. I can probably handle this tactfully - LH |
| 3.9 Lines 344-346: “In conclusion, the findings from this dataset underscore a significant alignment with national trends indicating a rapid increase in the number of TGD adults actively pursuing gender-affirming care.” I recommend re-writing this sentence. Unintentionally, by using language such as “rapid increase,” this may fuel arguments around social contagion and push the narrative that the number of TGD people is increasing rapidly, and the number of folks seeking gender-affirming care is rapidly increasing. It is clear that this is not your intention, but I immediately had this thought when reading this. | Thank you for catching this. We have reworded this sentence by replacing ‘rapid’ and adding more text. |
